# Associations between Bone Mineral Density and Longitudinal Changes of Vertebral Bone Marrow and Paraspinal Muscle Composition Assessed Using MR-Based Proton Density Fat Fraction and T2* Maps in Patients with and without Osteoporosis

**DOI:** 10.3390/diagnostics12102467

**Published:** 2022-10-12

**Authors:** Florian Tilman Gassert, Leander Glanz, Christof Boehm, Jonathan Stelter, Felix Gerhard Gassert, Yannik Leonhardt, Georg C. Feuerriegel, Markus Graf, Markus Wurm, Thomas Baum, Rickmer F. Braren, Benedikt J. Schwaiger, Marcus R. Makowski, Dimitrios Karampinos, Alexandra S. Gersing

**Affiliations:** 1 Department of Radiology, Klinikum Rechts der Isar, School of Medicine, Technical University of Munich, 81675 Munich, Germany; 2 Department of Trauma Surgery, Klinikum Rechts der Isar, School of Medicine, Technical University of Munich, 81675 Munich, Germany; 3 Department of Neuroradiology, Klinikum Rechts der Isar, School of Medicine, Technical University of Munich, 81675 Munich, Germany; 4 German Cancer Consortium (DKTK), Partner Site Munich, DKFZ Heidelberg, 68120 Heidelberg, Germany; 5 Department of Neuroradiology, Ludwig-Maximilians-University, 80333 Munich, Germany

**Keywords:** osteoporosis, magnetic resonance imaging, spine, bone marrow

## Abstract

Background: Proton-density fat fraction (PDFF) and T2* of the vertebrae, as well as the cross-sectional area (CSA) of the paraspinal musculature (PSM), have been suggested as biomarkers for bone fragility. The aim of this study was to longitudinally assess changes in PDFF, T2* and CSA of the PSM over 6 months in patients with and without osteoporosis. Methods: Opportunistic bone mineral density (BMD) measurements (BMD < 120 mg/cm^3^) were obtained from a CT acquired during the clinical routine work up in osteoporotic/osteopenic patients (*n* = 29, mean age 72.37 ± 10.12 years, 16 women). These patients were frequency-matched for age and sex to subjects with normal BMD values (*n* = 29). All study patients underwent 3T MR imaging at baseline and 6-month follow up, including spoiled gradient echo sequences for chemical shift encoding-based water-fat separation, from which T2* and PDFF values of the lumbar spine and the PSM were obtained. Moreover, the CSA of the PSM was assessed longitudinally. Changes in T2*, PDFF and CSA over 6 months were calculated for the vertebrae and PSM and associations with baseline BMD values were assessed. Results: The change in CSA of the PSM over 6 months was significantly lower in the osteoporotic/osteopenic group (−91.5 ± 311.7 mm^2^), compared to the non-osteoporotic group, in which the CSA increased (29.9 ± 164.0 mm^2^, *p* = 0.03). In a further analysis, patients with higher vertebral PDFF at baseline showed a significantly stronger increase in vertebral T2*, compared to those patients with lower vertebral PDFF at baseline (0.9 ± 1.6 ms vs. 0.0 ± 1.8 ms, *p* = 0.04). Moreover, patients with higher PSM PDFF at baseline showed a significantly stronger increase in vertebral T2*, compared to those patients with lower PSM PDFF at baseline (0.9 ± 2.0 ms vs. 0.0 ± 1.3 ms, *p* = 0.03). Conclusion: The PSM CSA decreased significantly longitudinally in patients with osteoporosis/osteopenia, compared to those without. Additionally, higher vertebral and PSM PDFF at baseline were associated with stronger changes in vertebral bone marrow T2*. Therefore, longitudinal PDFF and T2* mapping may be useful quantitative radiation-free tools for the assessment and prediction of muscle and bone health in patients with suspected osteoporosis/osteopenia.

## 1. Introduction

Muscle and bone are interconnected musculoskeletal units both in biomechanical and biochemical aspects [1,2]. With aging, the musculoskeletal system degenerates, leading to a reduction in strength and function of muscle and bone. Within the vertebral bone, the degenerative process induces a decrease in bone mineral density (BMD) and an increase in the bone marrow fat fraction (BMFF), leading to an increased risk of vertebral fractures and subsequent complications [3,4]. Within the muscle, fatty infiltration was reported to be the main factor in decreasing muscle strength [5]. Moreover, it has previously been shown that vertebral compression fractures are associated with a lower cross sectional area (CSA) in paraspinal musculature (PSM) [6] and that age- and sex-specific paraspinal muscle fat infiltration predicts the vertebral lumbar BMD [7]. Yet, in this previous study, muscle fat infiltration was assessed using axial T2-weighted MR images of the lumbar spine with only one echo time, which is limited in quality, since this sequence does not allow the direct quantitative assessment of muscle or vertebral bone marrow fat but rather provides a relative fraction, compared to a region of interest in an adjacent area of subcutaneous pure fat only.

Previously chemical shift encoding-based fat quantification was introduced as a MR-based technique that has been applied for the assessment of muscle composition [8], since this technique allows the measurement of the MR-based proton density fat fraction (PDFF), as well as vertebral T2* values. Although MR-based mapping techniques have shown to be useful for the assessment of various body tissues, including, e.g., T2* mapping in order to assess iron depositions within the brain [9] or T2 mapping for assessment of cartilaginous tissue [10], chemical shift encoding-based fat quantification has been considered to be a promising tool for the assessment of the fatty infiltration of muscles and liver [11], as well as for the assessment of vertebral bone marrow water-fat composition [12,13,14]. In contrast to the computed tomography (CT), during the MR-based assessment of vertebral bone marrow water-fat composition, the patient is not exposed to radiation [15,16]. Previous studies have demonstrated an inverse correlation between the lumbar BMFF and BMD [17,18,19], suggesting associations between fatty muscle infiltration and osteopenia/osteoporosis. A further study investigated the associations between lumbar vertebral bone marrow composition and paraspinal muscle composition and revealed significant correlations between the PDFF of paraspinal muscle and vertebral bone marrow compartments in postmenopausal women in a cross-sectional study [20].

Vertebral T2* values allow the assessment of the trabecular microstructure and have shown to enable the radiation-free differentiation between patients with low-energy osteoporotic and high-energy traumatic vertebral fractures, emphasizing its potential as a biomarker for the assessment of bone fragility and fracture risk [21]. Moreover, a previous assessment of the MR-based PDFF demonstrated that vertebral PDFF values were significantly higher in osteoporotic/osteopenic patients with vertebral fractures, compared to osteoporotic/osteopenic patients without vertebral fractures [22]. This study also demonstrated that patients with low-energy osteoporotic/osteopenic fractures had a significantly higher PDFF, compared to patients with high-energy traumatic fractures, also indicating PDFF potentially serves as a biomarker for bone fragility [22]. Yet, these previous studies only assessed these parameters cross-sectionally.

The aim of this study was, therefore, to longitudinally assess quantitative changes in vertebral and PSM PDFF, vertebral T2* and PSM CSA over 6 months in patients with and without osteopenia/osteoporosis.

## 2. Methods

### 2.1. Patient Selection and Study Design

Between January 2018 and April 2020, 29 patients with osteopenia/osteoporosis, assessed using opportunistic BMD measurements [23,24] measured from a CT scan acquired as part of the diagnostic work up in clinical routine (BMD < 120 mg/cm^3^), were recruited for this study. These patients were frequency-matched for age and sex with 29 patients with normal BMD values (BMD ≥ 120 mg/cm^3^). Exclusion criteria were contraindications for MR imaging (e.g., pacemaker) (*n* = 1), pregnancy (*n* = 1), vertebral fractures (*n* = 3) and metal implants (*n* = 2). None of the selected patients received osteoporosis treatment, neither at baseline nor at follow up. At baseline and 6-month follow-up, all included patients were scanned with a specific MR imaging protocol described below. The study was approved by the local institutional review board (Institutional Review Board of Technical University Munich (2022-433-S-SR; 11.08.2022) and all patients gave written and informed consent prior to their participation in the study.

### 2.2. Computed Tomography and BMD Measurements

CT images were acquired using either a dual-layer dual-energy CT (IQon Spectral CT, Philips Healthcare, Amsterdam, The Netherlands) or a multislice detector CT (MDCT) (Philips iCT 256, Philips Healthcare). All CT scans were obtained in the craniocaudal direction with the patient in a supine position, 120 kV, and an adaptive tube load with a maximum of 250 mAs. BMD values were derived from asynchronously calibrated quantitative CT examinations, as described previously [22]. In brief, mean Hounsfield unit (HU) values were derived from circular ROIs in all lumbar vertebrae in representative slices using the IDS7 PACS (Sectra AB, Linkoeping, Sweden). BMD values were then calculated from the average HU values with conversion equations derived from asynchronous calibration, as described previously [22,25]. A random sample of 10 subjects was chosen and reanalyzed by the same radiologist after 4 weeks in order to calculate the intrareader reproducibility.

### 2.3. Magnetic Resonance Imaging Measurements

Two 3T-MRI systems (an Ingenia, Philips Healthcare and an Elition, Philips Healthcare) were used for the examination of the abdomen, including the lumbar spine. The patients were placed in supine position and a combination of anterior and posterior coil arrays was used. For the acquisition, a six-echo 3D multi-echo gradient-echo sequence was used to acquire all echoes in a single TR, using bipolar gradients. To reduce the scan time a Compressed SENSE factor of 4 was employed. The combination of CS and SENSE, as well as the reconstruction, were based on the vendor’s implementation (Compressed SENSE, Philips Healthcare), as previously shown in [26].

The six echoes were acquired with an axial acquisition, using the following parameters: repetition time TR/ first echo time TE1/ echo time step ΔTE = 7.8/1.35/1.1 ms, field of view (FOV) = 300 × 400 × 150 mm^3^, acquisition voxel size = 2 × 3 × 6 mm^3^, reconstruction voxel size = 1.13 × 1.13 × 6 mm^3^, receiver bandwidth = 1678 Hz/pixel, frequency direction = anterior/posterior (A/P), 1 average, scan time = 9.3 s, using a combination of a 16-channel torso coil array and an inbuilt table posterior 12-channel coil array. To minimize T1-bias effects a flip angle of 3° was used [27].

Complex multi-echo gradient-echo images were provided as input to the fat quantification routine provided by the vendor (mDixon Quant, Philips Healthcare), as described previously [28]. The resulting PDFF map represents the ratio of the fat signal over the sum of fat and water signals. PDFF and T2* maps were extracted.

### 2.4. Paraspinal Muscle and Vertebra Segmentation

**Figure 1:**
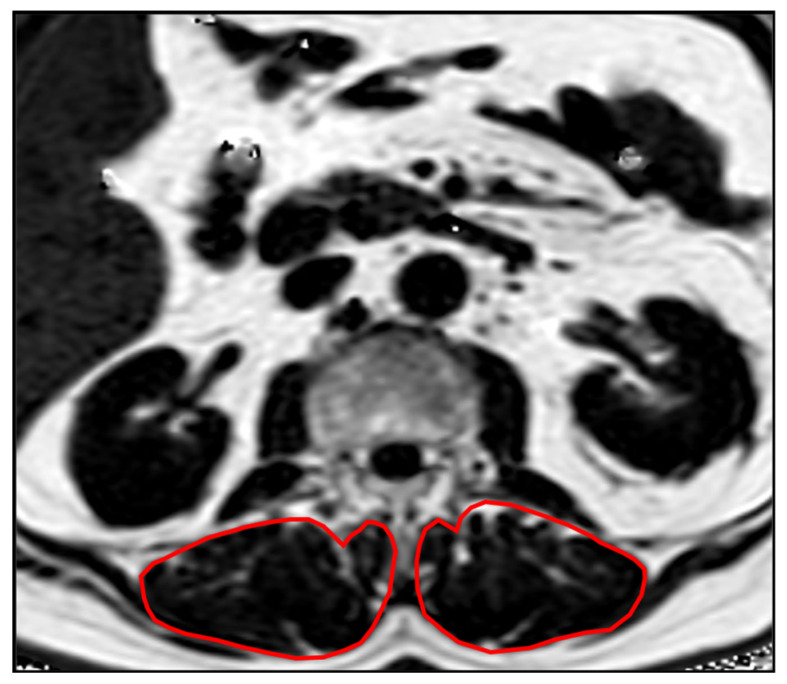
Example PDFF map showing the segmentations of the PSM.

Segmentations of the lumbar vertebrae and the paraspinal muscles were performed manually by a radiologist (F.T.G., 5 years of experience in musculoskeletal imaging) on the PDFF and T2* maps using the IDS7 PACS (Sectra AB, Linkoeping, Sweden). Beginning in vertebra L1, 5 slices were segmented. The paraspinal musculature was segmented bilaterally and values of both sides were averaged (Figure 1). Segmentations were reviewed by a board-certified radiologist (A.S.G. with 9 years of experience in musculoskeletal imaging).

The mean vertebral and PSM PDFF and vertebral T2* values of each patient were calculated as the average of the values derived from the five slices. The changes of PDFF and T2* values of both the lumbar spine and the PSM were calculated as the difference between the follow-up values after 6 months and the baseline values.

A random sample of 10 subjects was chosen and reanalyzed by the same radiologist in order to calculate the intrareader reproducibility error of the PDFF values.

### 2.5. Statistical Analysis

Statistical analysis was performed using SPSS version 25 software (IBM, New York, USA). A two-sided 0.05 level of significance was used. The Kolmogorov–Smirnov analysis exhibited no significant difference from a normal distribution for all, PDFF, T2*, and BMD values (*p* > 0.1). Pearson correlation coefficient was used to assess the correlations between the change of the vertebral PDFF, the change of the PSM PDFF, the change of the vertebral T2* and the change of the CSA in the PSM longitudinally over 6 months. A student’s t-test was used to analyze differences in vertebral and PSM PDFF, vertebral T2* and PSM CSA between patients with and without osteopenia/osteoporosis, as well as between subgroups. For subgroup analysis, an individual cut-off was chosen for baseline parameters vertebral PDFF and vertebral T2*, dividing all patients in two equal-sized groups (*n* = 29). Afterwards vertebral and PSM PDFF, as well as vertebral T2* changes, were assessed for these subgroups, respectively.

Intra-rater reproducibility for BMD, T2* and PDFF values were assessed by calculating the intraclass correlation coefficient (ICC).

### 2.6. Results

The average age of all patients included in this study was 71.56 ± 9.54 years (Table 1). Mean BMD was significantly lower in the osteoporotic/osteopenic group (92.7 ± 19.2 mg/cm^3^), compared to the non-osteoporotic/osteopenic group (162.1 ± 32.5 mg/cm^3^, *p* < 0.001). At baseline, mean vertebral PDFF of the lumbar spine was significantly higher in osteoporotic/osteopenic patients, compared to non-osteoporotic/non-osteopenic patients (41.6 ± 9.3% vs. 49.7 ± 14.7%, *p* = 0.01). Additionally, the vertebral T2* values of the lumbar spine were significantly higher in osteoporotic/osteopenic patients, compared to non-osteoporotic/non-osteopenic patients (9.9 ± 3.0 ms vs. 8.1 ± 2.3 ms, *p* = 0.008). Both, the mean PSM PDFF was higher in osteoporotic/osteopenic patients, compared to non-osteoporotic/non-osteopenic patients, yet this finding remained a statistical trend (8.2 ± 10.3% vs. 4.7 ± 4.4%, *p* = 0.10). The mean CSA of the PSM showed no significant difference between the two groups at baseline (1359.7 ± 440.0 mm^2^ vs. 1245.7 ± 316.9 mm^2^, *p* = 0.27).

Averaged over all patients of both groups, the vertebral PDFF increased by 3.1 ± 9.8% and the PSM PDFF increased by 0.3 ± 2.4% longitudinally over 6 months. Over all patients, vertebral T2* values increased by 0.7 ± 2.0 ms and the CSA of the PSM decreased by 15.2 ± 235.8 mm^2^ longitudinally over 6 months.

**Figure 2:**
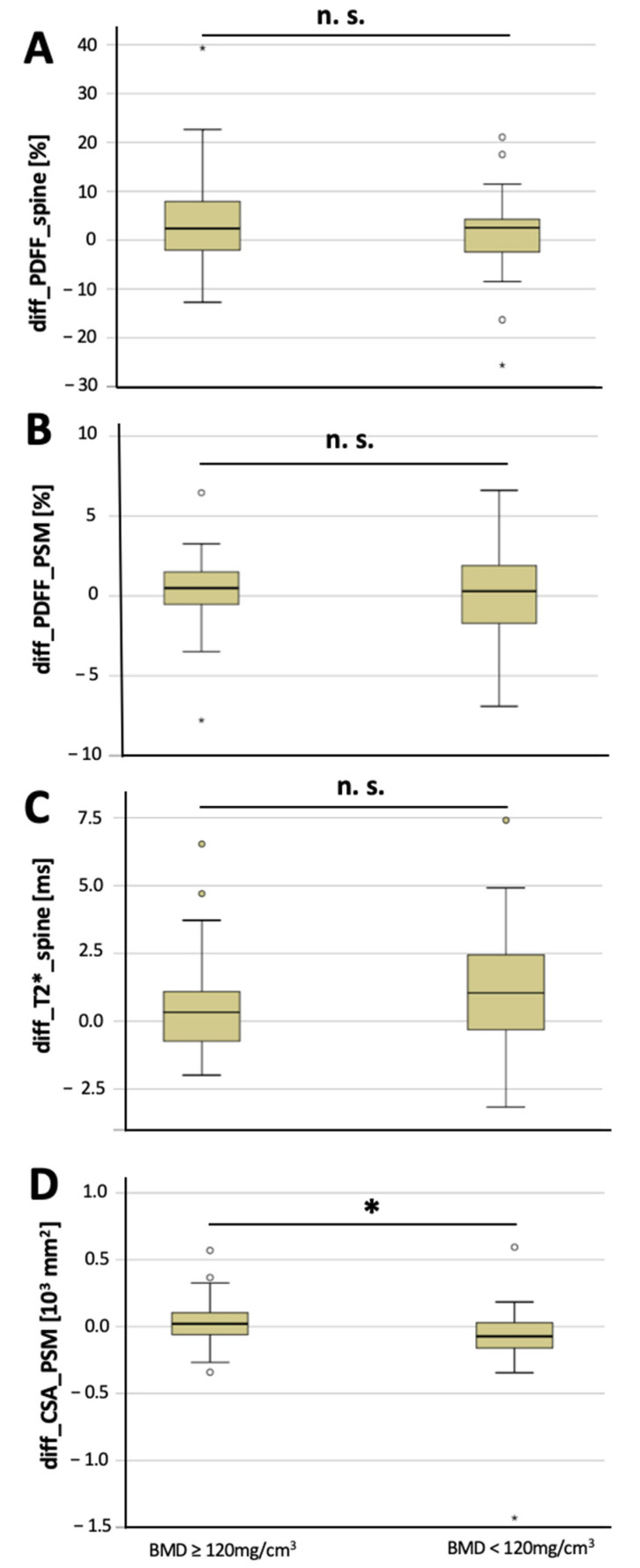
Longitudinal changes over 6 months in non-osteoporotic/osteopenic patients (≥120 mg/cm^3^) and in osteoporotic/osteopenic patients (BMD < 120 mg/cm^3^). Changes in vertebral PDFF (**A**, diff_PDFF_spine), changes in PSM PDFF (**B**, diff_PDFF_PSM), changes in vertebral T2* values (**C**, diff_T2*_spine) and changes in CSA of the PSM (**D**, diff_CSA_PSM) between baseline and 6-month follow-up (asterisks indicate significant difference). Boxplots: ° = outlier; * = extreme outlier.

**Figure 3:**
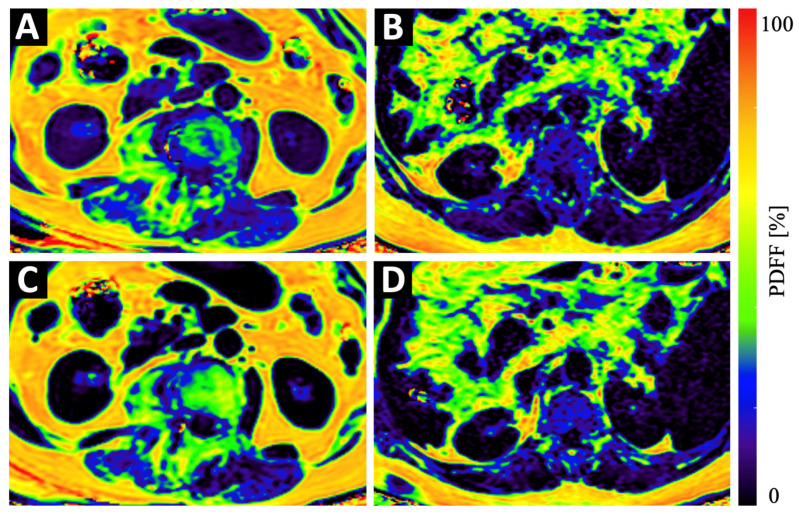
Example color-coded PDFF maps of a 84-year old osteopenic (BMD = 107 mg/cm^3^) female patient (**A**,**C**) and a 69-year old male patient with normal BMD values (123 mg/cm^3^) (**B**,**D**) at baseline (**A**,**B**) and 6 months follow-up (**C**,**D**). Although the maps of the osteopenic patient (**A**,**C**) show a strong increase in PDFF in vertebral bone and in the PSM, the increase in the maps of the non-osteopenic/osteoporotic patient (**B**,**D**) show only a very subtle increase in PDFF.

The CSA of the PSM decreased significantly longitudinally over 6 months in osteoporotic/osteopenic patients (−91.5 ± 311.7 mm^2^), compared to the non-osteoporotic/osteopenic patients (29.9 ± 164.0 mm^2^, *p* = 0.03; Figure 2), suggesting an accelerated decrease in CSA of the PSM longitudinally in patients with osteoporosis/osteopenia. Moreover, vertebral T2* value increase was greater in the osteoporotic/osteopenic patients, compared to the non-osteoporotic/osteopenic patients, yet this difference did not reach the level of significance (1.0 ± 2.4 ms vs. 0.5 ± 1.7 ms, *p* = 0.09). There was no significant difference found between the osteoporotic/osteopenic patients, compared to the non-osteoporotic/osteopenic patients regarding the longitudinal change in vertebral PDFF (4.3 ± 10.2 ms vs. 1.0 ± 9.0 ms, *p* = 0.15) and PSM PDFF (0.4 ± 2.2 ms vs. 0.0 ± 2.9 ms, *p* = 0.51) over 6 months (Figure 3).

In a further analysis, all patients were split into two equal-sized groups, depending on the baseline PDFF of the lumbar vertebrae (cut-off: 45.0%). In patients with a higher vertebral PDFF at baseline, the vertebral T2* value increase longitudinally over 6 months was significantly higher, compared to the T2* value increase in patients with lower vertebral PDFF at baseline (0.9 ± 1.6 ms vs. 0.0 ± 1.8 ms, *p* = 0.04) (Table 2). Moreover, when splitting the patients into two equal-sized groups, depending on the baseline PDFF of the PSM (cut-off: 4.00 %;), patients with higher baseline PSM PDFF showed a significantly higher increase in vertebral T2*, compared to those with lower baseline PSM PDFF (0.9 ± 2.0 ms vs. 0.0 ± 1.3 ms, *p* = 0.03).

A further analysis showed that when splitting the patients into two equal-sized groups depending on the baseline vertebral T2* values (cut-off: 9.0 ms), change in vertebral PDFF was significantly greater longitudinally over 6 months in patients with higher baseline vertebral T2* values, compared to those with lower baseline vertebral T2* values (7.1 ± 10.1% vs. 0.8 ± 10.0%, *p* = 0.02) (Table 2).

The intrareader (ICC, 0.979 [95% CI, 0.965–0.999]) agreement for BMD, as well as for T2* (ICC, 0.981 [95% CI, 0.968–0.999]) and for PDFF (ICC, 0.976 [95% CI, 0.959–0.999]) measurements were excellent.

## 3. Discussion

In this study, we evaluated longitudinal changes in PSM CSA, as well as vertebral and PSM PDFF and vertebral T2* between patients with and without osteoporosis/osteopenia, as well as longitudinal associations between these parameters. The decrease in PSM CSA was significantly greater in patients with osteoporosis/osteopenia compared to those without osteoporosis/osteopenia. Moreover, a statistical trend was found towards higher increase in T2* in patients with osteoporosis/osteopenia, compared to those without osteoporosis/osteopenia longitudinally over 6 months. Additionally, patients with higher PSM PDFF at baseline, as well as patients with higher vertebral PSM, showed a higher increase in vertebral T2* over 6 months, suggesting an accelerated longitudinal development of bone fragility.

Chemical shift-encoding based water-fat MR imaging was previously assessed for the analysis of body fat distribution [29]. All derived parameters, PDFF and T2* of both the spine and the paraspinal musculature, as well as the cross-sectional area of the paraspinal musculature, have previously been evaluated in multiple cross-sectional studies. Additionally, while many studies examined PDFF or T2* of either spine or PSM, we investigated all parameters, as well as PSM CSA, in a single study in order to assess associations between the parameters.

As different types of tissue lead to variations in magnetic susceptibility, which lead to variations in T2* relaxation times, information on tissue composition may be obtained using T2*-measurements. In this context, T2*-measurements are sensitive to inhomogeneities caused by susceptibility differences shortening the T2* decay of both the water and fat components and subsequently leading to a rapid decay of the measured gradient echo signal with increasing echo time [30,31]. For the spine, those inhomogeneities are known to be present at the inter-surface of bone marrow and bone trabeculae [21]. It has already been demonstrated previously that T2* of vertebral bone depends on bone density and the microarchitectural trabecular structure, whereas T2* increases with decreasing bone density [32]. In a further study, T2* mapping of vertebral bone marrow enabled the differentiation between patients with low-energy osteoporotic and high-energy traumatic vertebral fractures, suggesting T2* being a potential biomarker for bone fragility [21]. There was no significant difference found in BMD between patients with low-energy osteoporotic and high-energy traumatic vertebral fractures in this previous study. The fact that higher vertebral T2* values at baseline, as well as a longitudinally higher, increase in vertebral T2* values were found in patients with osteoporosis/osteopenia, compared to those without osteoporosis/osteopenia underlines this hypothesis. Moreover, the results suggest that T2* may detect subtle changes in bone composition earlier than the CT-derived BMD measurements. Therefore, this may indicate that vertebral T2* values are an even better predictor for bone fragility than the BMD.

The vertebral PDFF derived from chemical shift encoding-based water-fat separation measures a density map of hydrogen protons, which can be attributed to fat. [33]. Thus, it allows for a relative precise estimation of the fat volume fraction of an examined tissue due to the almost equal relative proton densities of fat and water [34]. Therefore, PDFF has been proposed to be a useful non-invasive tool for the diagnosis of several diseases, e.g., hepatic steatosis [33,35], but also for the assessment of bone matrix changes [22,36,37,38,39,40,41].

Additionally, the PSM PDFF has been evaluated previously. One study evaluated fat volume and PDFF of the psoas muscle and the BMI in a longitudinal model of patients with cancer cachexia [42]. This previous study found that in the investigated cohort of 58 oncological patients, the psoas muscle fat infiltration measured by PDFF correlated with severity of weight loss. Additionally, a study by Zhao et al. showed that PSM and vertebral bone are interacting tissues as they found that PDFF of the erector spinae, multifidus, and psoas of subjects with normal bone density were all significantly lower, compared to those PDFF values assessed in subjects with osteoporosis/osteopenia [43].

Although the PDFF and T2* of the bone have previously been suggested to serve as potential biomarkers for bone fragility, the change of PDFF showed no significant difference between the patients with and without osteoporosis/osteopenia longitudinally over 6 months in this study. However, a higher baseline PDFF of both the spine and the PSM resulted in a stronger increase in T2* values, suggesting accelerated bone fragility in these patients [21].

This study has limitations. The sample size assessed in this study was fairly small. Therefore, future studies in larger study cohorts are needed in order to confirm the findings of this study. Moreover, neither data on the body mass index nor on chronic diseases was available. Future prospective studies are needed in order to assess associations between theses parameters and changes in PDFF and T2*. We chose CT-derived BMD values, since previous publications have demonstrated CT-derived BMD values to be the more accurate method for the estimation of the BMD, compared to DXA measurements. All patients included in the study underwent CT imaging as part of the clinical routine diagnostic work up and were, therefore, not exposed to additional radiation. Additionally, the time between baseline and the follow-up assessed in this study (6 months) is rather short, which might be one reason for no significant differences regarding the changes in vertebral PDFF and T2* between patients with and without osteoporosis/osteopenia. In this longitudinal data set, only axial MR slices were available, potentially causing partial voluming. Further studies with an image acquisition in a sagittal plane need to be performed in order to confirm the results.

In conclusion, the PSM CSA decreased significantly longitudinally over 6 months in patients with osteoporosis/osteopenia, compared to those without. Moreover, higher vertebral PDFF and PSM PDFF at baseline were associated with more severe bone fragility. Therefore, longitudinal PDFF and T2* mapping may be useful quantitative radiation-free tools for the assessment and prediction of muscle and bone health in patients with suspected osteoporosis/osteopenia.

## Figures and Tables

**Table 1:** Subject demographics and baseline values.

Parameter	All	Normal BMD	Osteoporosis/Osteopenia	*p*-Value
Men/Women	26/32	13/16	13/16	
Age (years)	71.6 ± 9.5	70.2 ± 10.3	73.0 ± 11.5	*p* = 0.48
Follow-up time (days)	183 ± 22	180 ± 16	186 ± 19	*p* = 0.73
Baseline BMD (mg/cm^3^)	137.1 ± 43.8	162.1 ± 32.5	92.7 ± 19.2	*p* < 0.001
Baseline vertebral PDFF (%)	45.7 ± 8.7	41.6 ± 9.3	49.7 ± 14.7	*p* = 0.01
Baseline vertebral T2* (ms)	9.0 ± 2.4	8.1 ± 2.3	9.9 ± 3.0	*p* = 0.008
Baseline PSM PDFF (%)	6.5 ± 5.1	4.7 ± 4.4	8.2 ± 10.3	*p* = 0.10
Baseline PSM CSA (mm^2^)	1302.7 ± 380.5	1359.7 ± 440.0	1245.7 ± 316.9	*p* = 0.008

Note—Values are given as mean ± standard deviation. *p*-values for the significance of differences between the osteoporotic/osteopenic patients and patients with normal BMD are listed in the very right column.

**Table 2:** Subgroup analysis.

Parameter	Cut-Off Value	>Cut-Off	<Cut-Off	*p*-Value
		diff_T2*_spine (ms)		
Baseline vertebral PDFF (%)	45.0	0.9 ± 1.6	0.0 ± 1.8	*p* = 0.04
Baseline PSM PDFF (%)	4.0	0.9 ± 2.0	0.0 ± 1.3	*p* = 0.03
		diff_PDFF_spine (%)		
Baseline vertebral T2* (ms)	9.0	7.1 ± 10.1	0.8 ± 10.0	*p* = 0.02

Note—Values are given as mean ± standard deviation. *p*-values for the significance of differences are listed in the very right column.

## Data Availability

The data presented in this study are available on request from the corresponding author. The data are not publicly available due to privacy.

## Abbreviations

PDFF	Proton-Density Fat Fraction
BMD	Bone mineral density
BMFF	Bone marrow fat fraction
CSA	Cross sectional area
MDCT	Multislice detector CT
HU	Hounsfield Unit
PSM	Paraspinal muscle
